# Two Spectroscopies in One: Interference of Circular Dichroism and Raman Optical Activity

**DOI:** 10.1002/anie.202011146

**Published:** 2020-10-19

**Authors:** Tao Wu, Guojie Li, Josef Kapitán, Jiří Kessler, Yunjie Xu, Petr Bouř

**Affiliations:** ^1^ Institute of Organic Chemistry and Biochemistry Flemingovo náměstí 2 16610 Prague Czech Republic; ^2^ Department of Chemistry University of Alberta Edmonton Alberta T6G 2G2 Canada; ^3^ Department of Optics Palacký University Olomouc 17. listopadu 12 77146 Olomouc Czech Republic

**Keywords:** chirality transfer, electronic circular dichroism, magnetic circular dichroism, polarized Raman scattering, resonance Raman optical activity

## Abstract

Previously, we and other laboratories have reported an unusual and strong Raman optical activity (ROA) induced in solvents by chiral dyes. Various theories of the phenomenon appeared, but they were not capable of explaining fully the observed ROA band signs and intensities. In this work, an analysis based both on the light scattering theory and dedicated experiments provides a more complete understanding. For example, double‐cell magnetic circular dichroism and magnetic ROA experiments with copper‐porphyrin complex show that the induced chirality is observed without any contact of the solvents with the complex. The results thus indicate that a combination of electronic circular dichroism (ECD) with the polarized Raman scattering is responsible for the effect. The degree of circularity of solvent vibrational bands is a principal molecular property participating in the event. The insight and the possibility to predict the chirality transfer promise future applications in spectroscopy, chemical analysis and polarized imaging.

## Graphical abstract

In a series of experiments and by a theoretical analysis we decipher Raman optical activity induced in achiral solvents as an interference of two phenomena, circular dichroism and polarized Raman scattering. Applications of this strong effect can be potentially found in analytical chemistry, spectroscopy, and imaging of biologically relevant systems.

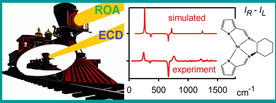

Interaction of light with chiral molecules is a fascinating topic, discovered already in early experiments of Faraday and Pasteur,^[1]^ followed much later by spectroscopy of vibrational circular dichroism (VCD),^[2]^ Raman optical activity (ROA) and many other techniques.^[3]^ Quite recently, for example, enhanced VCD was pointed out as a useful tool for studies of protein fibrils associated with neurodegenerative diseases,^[4]^ resonance ROA spectra were found sensitive to carotenoid aggregation^[5]^ or paramagnetic excited states of halogen gases.^[6]^

Also “chirality transfer” phenomena when chiral molecules make non‐chiral ones optically active attracted attention because of multiple aspects.^[7]^ The chirality induction provides a detailed insight into molecular interactions^[8]^ and is thus interesting for nanotechnology industry^[9]^ and analytical chemistry.^[10]^ For ROA, the effect can be surprisingly strong,[^7a^, ^7d^] which would potentially broaden application field of the technique, often hampered by low sensitivity and instrumental artifacts.^[11]^

Lately, for example, we reported such a strong effect for a nickel complex dissolved in a wide range of achiral organic solvents.^[12]^ The solvent optical activity was even larger than that of the complex or natural chirality in case of a chiral solvent. The effect could be linked to the absorption of the complex, which provides enhanced Raman/ROA signals in resonance or pre‐resonance with the excitation laser beam. Ab initio computations and a series of experiments excluded short‐distance interactions between the solute and the solvent as the primary source of the effect. An ad hoc “ring of fire” model was proposed, which explained most of the observed ROA band signs, but not band magnitudes. In particular, the predicted ROA to Raman signal ratio (so called circular intensity difference, *CID* ^[13]^) was too small.

The key observation explaining this inconsistency is that the strong chirality transfer/induction was observed for transition metal complexes, where the metals provide extremely large magnetic dipole moments in the *d‐d* electronic transitions.^[14]^ They can mix, for example, with vibrational ones generating enhanced VCD signal.^[15]^ In a resonance/pre‐resonance ROA experiment (where frequency of the laser light is close to that of an electronic transition) these complexes also lead to a strong electronic circular dichroism (ECD, differential absorption of left‐ and right‐circularly polarized light, CPL). This left/right CPL imbalance can be ultimately detected as an additional component of ROA (difference in intensities of right‐ and left‐CPL during Raman scattering).

Let us examine a common scattered circular polarized (SCP) backscattering ROA experiment^[16]^ in detail. The light enters the sample and the polarization difference is detected in the scattered light (Figure 1, also Figures S1 and S2 in Supporting Information). However, when the sample exhibits ECD, the incoming light polarizes even before (and also after) being scattered, for example, in a volume element schematically labeled *dl* in the Figure. Recorded ROA signal thus gains additional, sometime dominant component from ECD. We believe that this event has been ignored in previous literature.

**Figure 1:**
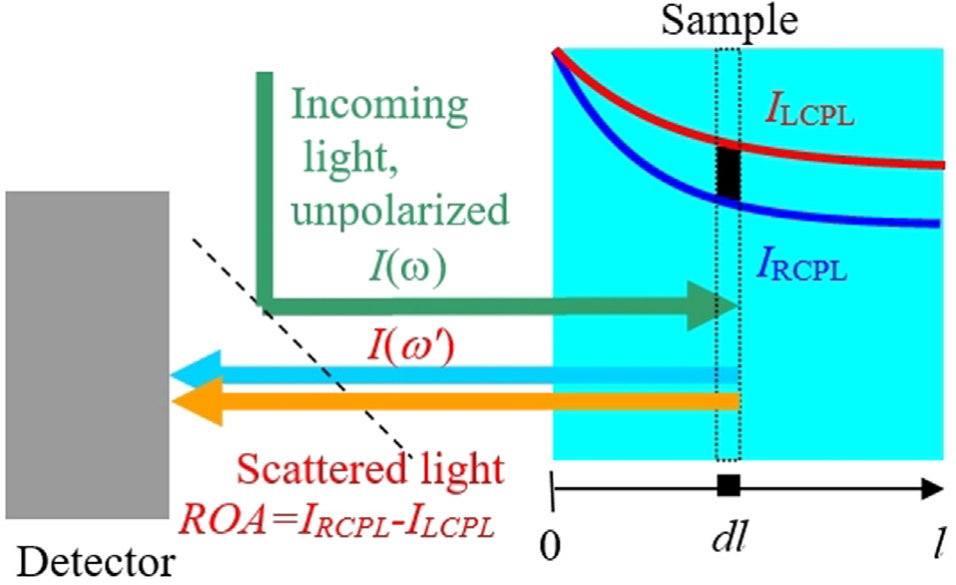
Geometry of the ROA experiment. When the sample contains a compound differently absorbing left‐ and right‐circularly polarized light, additional chirality arises when the light is travelling through it.

The interference of ECD with ROA is in practice a complicated event. The scattering occurs in a tiny volume (typically < ≈60 μL). The laser is focused in even smaller spot and most volume does not participate in the scattering. Samples absorbing the laser light may decompose, exhibit strong resonance Raman signal and fluorescence. Temperature‐induced variation of the refractive index can cause a “thermal lensing”. The light polarized through ECD passes through the solution, undergoes Raman‐scattering and further depolarizes.

Nevertheless, theoretical considerations do allow to estimate the extent of mixing of the ECD and ROA phenomena. Observed ratio of the ROA and Raman signals (under usual conditions, cf. Supporting Information) can be obtained as

(1) $$CID=\frac{I_{R}-I_{L}}{I_{R}+I_{L}}=\frac{\Delta \epsilon^{'}+DOC\Delta \epsilon }{4}cL$$

where Δ*ϵ* and Δ*ϵ′* are differential absorption indices of the excitation and scattered light, respectively, determining ECD intensity, *I_R_* and *I_L_* are detected intensities of right and left CPL, *c* is the concentration, *L* is optical path length, and *DOC* is the degree of circularity of each vibrational transition of the solvent.^[13]^

From (1) we see that *CID* has the potential to be relatively large compared to usual ROA experiments. Whereas typical *CID*s for organic molecules is about 10^−4^, the ECD “dissymmetry ratio, Δ*ϵ*/*ϵ*, is about 10^−3^. For metal *d*‐d of *f*‐*f* transitions this quantity can even approach one (!).[^12^, ^15^, ^17^]

The solvent thus enters formula (1) through the *DOC* parameter, which says how the scattered molecule “remembers” the initial circular polarization. *DOC* is also connected to Raman scattering depolarization ratios, or just to molecular polarizability changes under a particular vibration.

To verify the interference of ECD and ROA, we analyzed ROA and magnetic ROA spectra for model nickel and copper complexes, structures of which are plotted in Figure 2. Chloroform solutions of the first two **Ni** and **Cu** compounds provide ECD and ROA spectra plotted in Figure 3. As discussed before, the strongest ROA bands belong to the chloroform solvent.^[12]^ However, the two complexes behave differently. ECD signal of **Ni** is maximal at the excitation 532 nm wavelength, and it quickly diminishes within the scattered wavenumber range, i.e., |Δ*ϵ*′| < |Δ*ϵ*|. From formula (1) we see that the degree of circularity multiplying Δ*ϵ* will be most important in this case. Indeed, simulated signs and relative band intensities nearly copy the *DOC* values (Table S1), and in general agree with the experiment.

**Figure 2:**
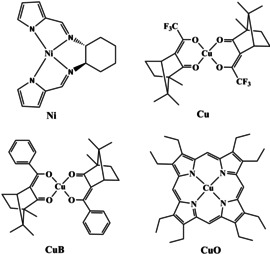
The four complexes investigated: (*R*,*R*)‐bis(pyrrol‐2‐ylmethyleneamine)‐cyclohexane nickel(II) (*R*‐**Ni**), *R*‐bis‐(trifluoroacetylcamphorato) copper(II) (*R*‐**Cu**), *R*‐bis‐(benzylcamphorato) copper(II) (*R*‐**CuB**), octaethylporphyrinato‐ copper(II) (**CuO**).

**Figure 3:**
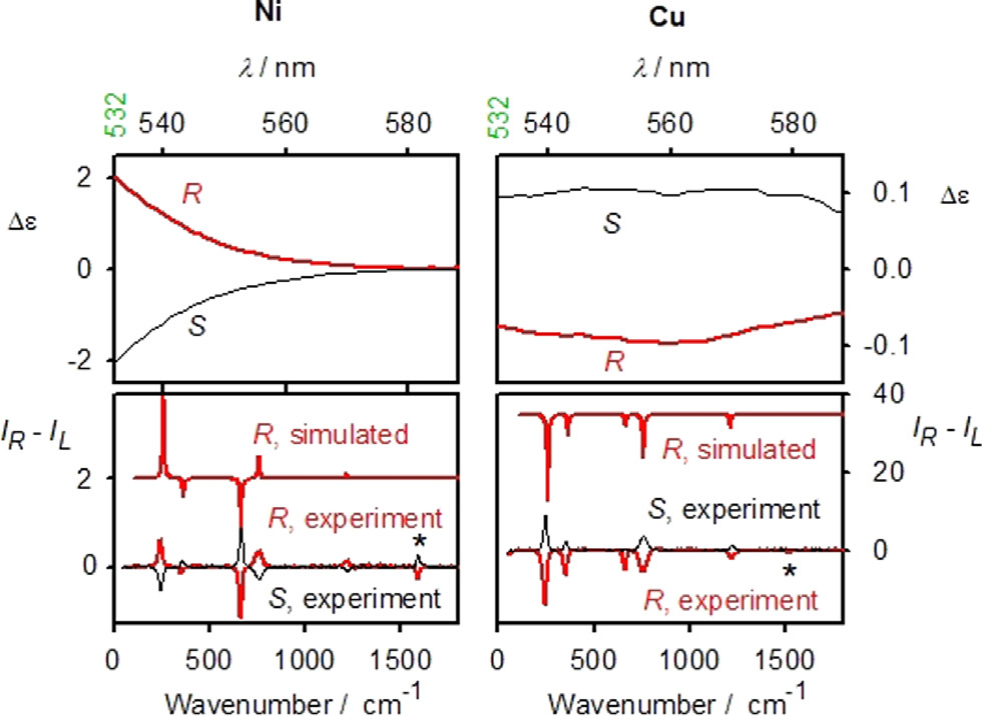
ECD (Δ*ϵ*, L mol^−1^ cm^−1^ top) and ROA (Δ*I*, bottom, arbitrary units) spectra of **Ni** and **Cu** solutions in CHCl_3_ within the region 0 to 1800 cm^−1^, relative to the 532 nm laser, the asterisk marks the strongest band coming from the complex itself.

On the other hand, for **Cu** the ECD intensity is about constant (Δ*ϵ*′≈Δ*ϵ*) in the scattering range, which results in single‐sign ROA (Figure 2, right, bottom). The (*RR*)‐**Cu** complex gives negative ECD and induced chloroform ROA of the same sign. Similarly, the *R*‐**CuB** complex induces negative chloroform ROA below 2000 cm^−1^; however the 3021 cm^−1^ band is positive, in accordance with ECD sign change (Figure S3).

Another illustrative proof of the ECD and ROA interference is provided by a double‐cell experiment, where the complex did not have any chemical contact with the solvent which exhibited induced ROA (Figure 4, top). Magnetic circular dichroism (MCD) was used instead of natural ECD, where the “chirality” could be conveniently changed by flipping the orientation of the magnet.

**Figure 4:**
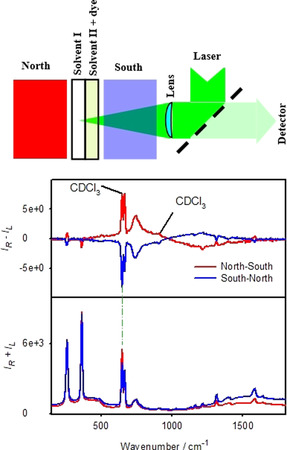
The magnetic ROA experiment (top, schematically), the complex (dye) is in a different cell than the investigated solvent. At the bottom, Raman (*I_R_*+*I_L_*) and ROA (*I_R_*−*I_L_*) spectra are plotted as obtained for two orientations of the magnet for solvent I=CDCl_3_, solvent II=**CuO** dissolved in CHCl_3_.

For the **CuO** complex dissolved in CHCl_3_ in ROA we can see not only induced ROA of chloroform, but also two bands of CDCl_3_, not in contact with the dye (Figure 4, bottom). MCD spectra are provided in Figure S4 and suggest that the dichroism of the scattered radiation is much larger than that of the impinging one (|Δ*ϵ*′| > |Δ*ϵ*|), which is consistent with the one‐sign induced ROA pattern below 900 cm^−1^. Around 560 nm (≈940 cm^−1^) the MCD sign changes; however high ROA noise makes it impossible to verify signs of induced ROA bands above this limit. Note also, that formula (1) would have to be modified to describe the double‐cell experiment, which goes beyond the scope of this work.

An analogous result was obtained when CDCl_3_ was replaced by ethanol. In this case at least one induced ROA ethanol band is visible, of the same sign as for CDCl_3_ (Figure S5). Also when the natural circular dichroism, instead of the magnetic one, is used as a source of the chirality, solvent ROA signal is induced without direct contact with the dye. This is shown in Figure 5 for *R*‐**Cu** and *R*‐**CuB**. The single‐sign ECD within 532–590 nm (0–1800 cm^−1^, Figure 3 and Figure S3) favors one‐sign ROA pattern. Because of the much larger absorption and ECD of **CuB** at 532 nm than for **Cu**, the induced ROA is also larger. In both cases, most ethanol bands can be recognized in ROA. Experimental details related to the synthesis of used compounds and all spectroscopic measurements are summarized in the Supporting Information.

**Figure 5:**
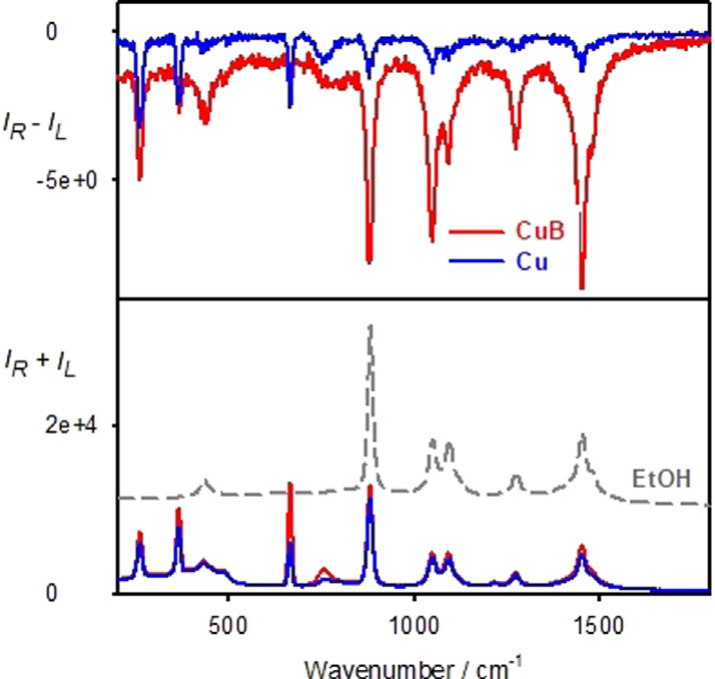
ROA (top) and Raman (bottom) spectra from the double‐cell experiment without magnet, solvent I=ethanol, solvent II=CHCl_3_ with *R*‐**Cu** (40 mM) or *R*‐**CuB** (10 mM) as the dye.

Circular dichroism of the chiral complexes thus appears as the dominant factor leading to the “transfer” of their chirality to the solvents. In ref. [12] we wrongly proposed chiral resonance Rayleigh scattering as the main mechanism. The Rayleigh scattering is present, but cannot explain the strength of measured ROA intensities. In both cases, chiral Rayleigh scattering and ECD, the excitation light is made chiral by the metal complex/dye, and further Raman‐scattered by the solvent. Therefore the former Rayleigh “ring of fire”^[12]^ model could correctly explain ROA band signs in case of the **Ni** complex.

In conclusion, we have analyzed the origin of the chirality transfer observed in resonance Raman optical activity measurements both with achiral and chiral systems, provided a theory that enabled prediction of the spectra, and verified it on several examples. The results confirmed the strength of the induction observed previously, and revealed an interesting intertwining of the ROA and ECD effects. The theoretical formula can be readily used to predict both observed signs and intensities. We find it important to recognize this phenomenon in chiral spectroscopic techniques, where it can produce unwanted artifacts. On the other hand, we believe that the effect itself has a potential to be used in many applications, such as in analytical chemistry, where the strength can conveniently overcome sensitivity limitations traditionally hampering chiral analysis.

## Conflict of interest

The authors declare no conflict of interest.

## Supporting information

As a service to our authors and readers, this journal provides supporting information supplied by the authors. Such materials are peer reviewed and may be re‐organized for online delivery, but are not copy‐edited or typeset. Technical support issues arising from supporting information (other than missing files) should be addressed to the authors.

Supplementary
