# Supplementary material for: Two Spectroscopies in One: Interference of Circular Dichroism and Raman Optical Activity
Source: Angew Chem Int Ed Engl. 2020 Oct 19;59(49):21895–8. doi: 10.1002/anie.202011146 (PMC7894312; doi:10.1002/anie.202011146)
Supplement: Supplementary file 1 — Supplementary [file ANIE-59-21895-s001.pdf]

Angewandte Chemie
Eine Zeitschrift der Gesellschaft Deutscher Chemiker

Supporting Information

Two Spectroscopies in One: Interference of Circular Dichroism and Raman Optical Activity

Tao Wu, Guojie Li, Josef Kapitán, Jiří Kessler, Yunjie Xu,* and Petr Bouř**

anie_202011146_sm_miscellaneous_information.pdf

Contents

Theory of ECD and ROA interference

Figure S1. Geometry of the backscattering SCP experiment.

Figure S2. Intensities of the left- and right-circularly polarized light components.

Experimental Section

Figure S3. ECD and chloroform-induced ROA spectra of *R*-**CuB** complex.

Figure S4. Magnetic CD of the **CuO** complex.

Figure S5. Magnetic ROA in the **CuO** $CHCl_3$/ethanol double-cell experiment.

Table S1. Chloroform Spectral Parameters

Theory of ECD and ROA interference

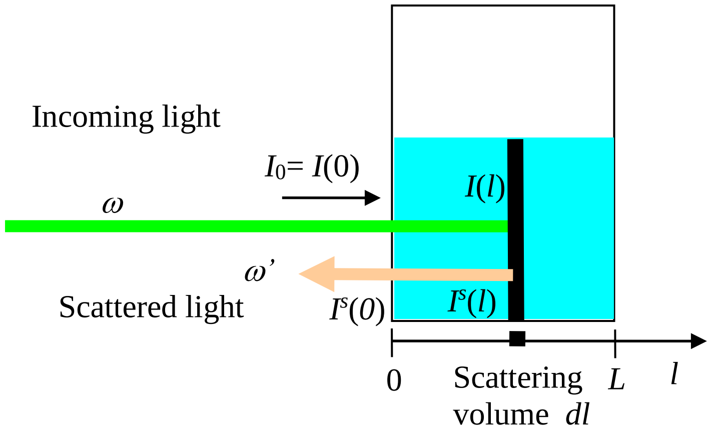

Figure S1. Geometry of the backscattering SCP experiment, incoming unpolarized light of frequency ω enters with intensity I_0 at l = 0, the scattered light with intensity I^s and frequency ω' leaves. The incoming light is a sum of left- and right-circularly polarized components, $I_0(0) = I_R(0) + I_L(0)$, where $I_R(0) = I_L(0) = I(0)/2$.

We describe situation in **Figure S1.** Let ε_L and ε_R be the absorption indices for left and right circular polarized light (LCPL, RCPL) of frequency ω, which enters the sample. The absorption index

$$\varepsilon = (\varepsilon_L + \varepsilon_R)/2 \qquad \text{(S1)}$$

and circular dichroism

$$\Delta\varepsilon = \varepsilon_L - \varepsilon_R . \qquad \text{(S2)}$$

Once entering the sample, the intensity diminishes, differently for the different polarizations, originally equally present in the excitation beam:

$$I_R(l) = I_0 e^{-\varepsilon_R cl}/2, \qquad \text{(S3)}$$
$$I_L(l) = I_0 e^{-\varepsilon_L cl}/2,$$

c is concentration and l is the path length. An example of the dependence of the intensities following the Lambert-Beer laws (3) on l is in **Figure S2**, plotted with rather unrealistically high $\Delta\varepsilon/\varepsilon$ ratio so that the difference is better seen. The difference $|I_R - I_L|$ is maximal at $l_{\max} = \frac{\ln(\varepsilon_L/\varepsilon_R)}{c\Delta\varepsilon} \cong \frac{1}{c\varepsilon}$, with $\Delta I_{\max} \cong \frac{I_0\Delta\varepsilon}{2e\varepsilon}$, where e = 2.71828 (**Comment 1**). For typical values encountered in experiment, $\varepsilon \sim 10^4$ L·mol^{-1}·cm^{-1}, $\Delta\varepsilon \sim 10$ L·mol^{-1}·cm^{-1} and $c \sim 10^{-4}$ mol/L, we obtain $l_{\max} \sim 1$ cm. This length is well comparable with cell dimensions (few mm) used in typical ROA measurement. The ratio $"CID" = \frac{I_R - I_L}{I_R + I_L}$, formally equal to the circular intensity difference, grows nearly linearly with l, $"CID" \sim \Delta\varepsilon cl/2$, and is positive for $\varepsilon_L > \varepsilon_R$. At $l = l_{\max}$, $"CID" \sim \Delta\varepsilon/(2\varepsilon)$, and it can grow further for $l > l_{\max}$.

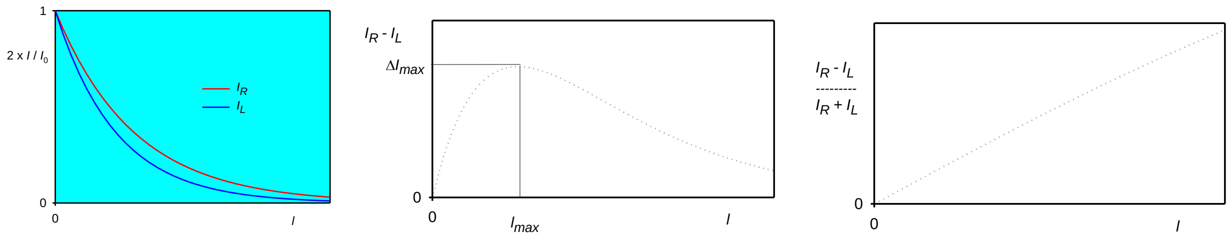

Figure S2. Intensities of the left- and right-circularly polarized light components as dependent on the path length *l*.

Backscattering from achiral solvent. Each volume element *Sdl* contributes to scattered intensities as

$$dI_R^s = aI_R(l)dl + bI_L(l)dl \qquad \text{(S4)}$$
$$dI_L^s = aI_L(l)dl + bI_R(l)dl\,,$$

where the polarization coefficients *a* and *b* determine polarization changes during the scattering and are related to the degree of circularity.[1] For brevity in (S4) and further on we omit the beam cross section (area) *S*, as this is just a constant. We additionally suppose that all volume contributes equally and neglect finite depth of field of the transfer optics. Re-arranging expressions (S4), we get Raman signal contributions,

$$dI_{Ram}^s(l) = dI_R^s + dI_L^s = (a+b)(I_R(l) + I_L(l))dl \qquad \text{(S5)}$$

The scattered light is also absorbed when passing back through the solvent. According to the Lambert-Beer law the intensity from volume *dl* at *l* = 0 is

$$dI_{Ram}^s(0) = (a+b)[I_R(l)e^{-\varepsilon_R'cl} + I_L(l)e^{-\varepsilon_L'cl}]dl\,, \qquad \text{(S6)}$$

where $\varepsilon_i' = \varepsilon_i(\omega')$ are absorption indices at the Raman scattering frequency. With a negligible error we can set $\varepsilon_L' \cong \varepsilon_R' = \varepsilon'$, $\varepsilon_L \cong \varepsilon_R = \varepsilon$, and combining (S6) with (S3) we get

$$dI_{Ram}^s(0) = (a+b)I_0 e^{-(\varepsilon'+\varepsilon)cl}dl\ . \qquad \text{(S7)}$$

Integration of (S7) over the whole volume gives

$$I_{Ram} = \int_{l=0}^{L} dI_{Ram}^s(0) = \frac{a+b}{c} \cdot \frac{1-e^{-(\varepsilon+\varepsilon')cL}}{\varepsilon+\varepsilon'} I_0\,. \qquad \text{(S8a)}$$

Formula (S8a) may be better understood when we investigate the limit of short path lengths/weak absorption when $2\varepsilon cL << 1$ and

$$I_{Ram} \sim (a+b)I_0 L\,. \qquad \text{(S8b)}$$

This means that the intensity does not depend on the concentration of the absorbing compound; all volume *L* is available for the scattering. This is the usual situation for non-resonance Raman experiments (concentration of the scattering medium may enter through coefficients *a* and *b*). For another extreme, long path lengths/strong absorption, $2\varepsilon cL >> 1$, we get

$$I_{Ram} \sim \frac{(a+b)I_0}{(\varepsilon+\varepsilon')c} \quad . \tag{S8c}$$

In this case only a effective penetration volume $L_{pen} = \frac{1}{(\varepsilon+\varepsilon')c}$ is available for the scattering, independent of the total volume/path length *L*.

Detected ROA, SCP. Unlike for Raman, for ROA we must distinguish different absorption indices for left and right CPL. Starting from eq. (S4) and following the Lambert-Beer law, we obtain ROA $dI^s_{ROA}(0) = dI^s_R(0) - dI^s_L(0)$ measurable at $l = 0$ as

$$dI^s_{ROA}(0) = [aI_R(l)dl + bI_L(l)dl]e^{-\varepsilon_R' cl} - [aI_L(l)dl + bI_R(l)dl]e^{-\varepsilon_L' cl} \tag{S9}$$

We use eq. (S3), $\Delta\varepsilon = \varepsilon_L - \varepsilon_R$, $\Delta\varepsilon' = \varepsilon_L' - \varepsilon_R'$, $\varepsilon' \cong \varepsilon_R'$, and $\varepsilon \cong \varepsilon_R$, so that

$$dI^s_{ROA}(0) = (a + be^{-\Delta\varepsilon cl} - ae^{-(\Delta\varepsilon+\Delta\varepsilon')cl} - be^{-\Delta\varepsilon' cl})I_0 e^{-(\varepsilon'+\varepsilon)cl} dl/2 \tag{S10}$$

and obtain the integral value

$$I_{ROA} = \int_{l=0}^{L} dI^s_{ROA}(0) = \left(a\frac{1-e^{-(\varepsilon'+\varepsilon)cL}}{\varepsilon'+\varepsilon} + b\frac{1-e^{-(\varepsilon'+\varepsilon+\Delta\varepsilon)cL}}{\varepsilon'+\varepsilon+\Delta\varepsilon} - a\frac{1-e^{-(\varepsilon'+\varepsilon+\Delta\varepsilon+\Delta\varepsilon')cL}}{\varepsilon'+\varepsilon+\Delta\varepsilon+\Delta\varepsilon'} - b\frac{1-e^{-(\varepsilon'+\varepsilon+\Delta\varepsilon')cL}}{\varepsilon'+\varepsilon+\Delta\varepsilon'}\right)\frac{I_0}{2c} \quad . \tag{S11}$$

In common experiments $\Delta\varepsilon \ll \varepsilon$, $\Delta\varepsilon' \ll \varepsilon'$, and $(\Delta\varepsilon'+\Delta\varepsilon)cL \ll 1$, which provides

$$I^{SCP}_{ROA} = [a(\Delta\varepsilon+\Delta\varepsilon') + b(\Delta\varepsilon'-\Delta\varepsilon)]\frac{1-e^{-(\varepsilon'+\varepsilon)cL}[cL(\varepsilon'+\varepsilon)+1]}{2(\varepsilon'+\varepsilon)^2 c} I_0 . \tag{S12}$$

Dividing (S12) by (S8a) we obtain measured *CID*,

$$CID = \frac{a(\Delta\varepsilon+\Delta\varepsilon') + b(\Delta\varepsilon'-\Delta\varepsilon)}{a+b}\frac{1-e^{-(\varepsilon'+\varepsilon)cL}[cL(\varepsilon'+\varepsilon)+1]}{2(\varepsilon'+\varepsilon)(1-e^{-(\varepsilon'+\varepsilon)cL})} . \tag{S13}$$

For weak absorption, $cL(\varepsilon'+\varepsilon) \ll 1$ (cf. **Comment 2** for details), and

$$CID \cong \frac{a(\Delta\varepsilon+\Delta\varepsilon') + b(\Delta\varepsilon'-\Delta\varepsilon)}{a+b}\frac{cL}{4} . \tag{S14a}$$

In case of strong absorption, $cL(\varepsilon'+\varepsilon) \gg 1$, and

$$CID = \frac{a(\Delta\varepsilon+\Delta\varepsilon') + b(\Delta\varepsilon'-\Delta\varepsilon)}{2(a+b)(\varepsilon'+\varepsilon)} . \tag{S14b}$$

Relation to molecular properties. For a sample consisting of chiral absorbing dye dissolved in a solvent, the absorption indices ($\Delta\varepsilon$, $\Delta\varepsilon'$, ε and ε') are functions of the dye, whereas the depolarization coefficient a and b depend on transition polarizabilities of the solvent. To find a and b, let us consider backscattering of LCPL on one molecule. Using the Stokes parameters,[1] for the excitation light, up to a constant, $S_0 = I_0$ and $S_3 = I_R - I_L = -I_0$. For the backscattered light (ref. [1], Eq. 3.5.3, considering only real symmetric polarizability $\boldsymbol{\alpha}$)

$$S_0' = \frac{K}{2}(\alpha_{xx}^2 + 2\alpha_{xy}^2 + \alpha_{yy}^2)I_0 \qquad \text{(S15)}$$
$$S_3' = K(\alpha_{xx}\alpha_{yy} - \alpha_{xy}^2)I_0$$

where K is a constant, and the z axis goes along l. Using isotropic averages $\left\langle \alpha_{xx}^2 \right\rangle = \left\langle \alpha_{yy}^2 \right\rangle = \frac{1}{15}(\alpha_{\alpha\alpha}\alpha_{\beta\beta} + 2\alpha_{\alpha\beta}\alpha_{\alpha\beta})$, $\left\langle \alpha_{xx}\alpha_{yy} \right\rangle = \frac{1}{15}(2\alpha_{\alpha\alpha}\alpha_{\beta\beta} - \alpha_{\alpha\beta}\alpha_{\alpha\beta})$, and $\left\langle \alpha_{xy}^2 \right\rangle = \frac{1}{30}(3\alpha_{\alpha\beta}\alpha_{\alpha\beta} - \alpha_{\alpha\alpha}\alpha_{\beta\beta})$ we get

$$S_0' = \frac{K}{30}(\alpha_{\alpha\alpha}\alpha_{\beta\beta} + 7\alpha_{\alpha\beta}\alpha_{\alpha\beta})I_0 \qquad \text{(S16)}$$
$$S_3' = \frac{K}{6}(\alpha_{\alpha\alpha}\alpha_{\beta\beta} - \alpha_{\alpha\beta}\alpha_{\alpha\beta})I_0$$

The backscattered intensities are $I_0' = I_R' + I_L' = S_0'$, $I_R' - I_L' = S_3'$, so that

$$I_R' = (S_3' + S_0')/2 = (3\alpha_{\alpha\alpha}\alpha_{\beta\beta} + \alpha_{\alpha\beta}\alpha_{\alpha\beta})\frac{KI_0}{30} \qquad \text{(S17)}$$
$$I_L' = (S_0' - S_3')/2 = (6\alpha_{\alpha\beta}\alpha_{\alpha\beta} - 2\alpha_{\alpha\alpha}\alpha_{\beta\beta})\frac{KI_0}{30}.$$

Comparing (S17) with (S4), we see that $b \sim 3\alpha_{\alpha\alpha}\alpha_{\beta\beta} + \alpha_{\alpha\beta}\alpha_{\alpha\beta}$ and $a \sim 6\alpha_{\alpha\beta}\alpha_{\alpha\beta} - 2\alpha_{\alpha\alpha}\alpha_{\beta\beta}$. Defining the degree of circularity $DOC = -S_3'/S_0'$ (cf. also eq. 2.123 in ref. [2]), we can write

$$DOC = 5\frac{\alpha_{\alpha\beta}\alpha_{\alpha\beta} - \alpha_{\alpha\alpha}\alpha_{\beta\beta}}{\alpha_{\alpha\alpha}\alpha_{\beta\beta} + 7\alpha_{\alpha\beta}\alpha_{\alpha\beta}} = \frac{a-b}{a+b} \qquad \text{(S18)}$$

Alternatively, we can relate a and b to the reversal coefficient (ref. [3], eq. 3.5.15 in ref. [1] or eq. 2.122 in ref. [2])

$$R = I_L'/I_R' = \frac{a}{b} = \frac{6\alpha_{\alpha\beta}\alpha_{\alpha\beta} - 2\alpha_{\alpha\alpha}\alpha_{\beta\beta}}{3\alpha_{\alpha\alpha}\alpha_{\beta\beta} + \alpha_{\alpha\beta}\alpha_{\alpha\beta}} \qquad \text{(S19)}$$

Eq. (S14a), for example, can be written as

$$CID \cong \frac{\Delta\varepsilon' + DOC\Delta\varepsilon}{4}cL. \qquad \text{(S20)}$$

The most important parameter determining solvent induced chirality is thus the degree of circularity.

To think about the result, it might be useful to consider solvent of a spherical symmetry (e.g. tetrachloromethane) where $\alpha_{\alpha\beta} = \alpha\delta_{\alpha\beta}$, *R* = 0 and $DOC = -1$. In this case, LCPL is after scattering totally converted to RCPL. From (S20) we see that observed *CID* is then just proportional to ECD difference between the scattered and incoming light. For $\Delta\varepsilon = \Delta\varepsilon'$ we get the obvious result that circular polarization due to the ECD of the excitation beam is annulated by ECD of the scattered radiation, because the path length and concentrations are the same.

Detected ROA, ICP. When the sample is irradiated exclusively with L or R CPL, intensities (S3) at point l become

$$I_R(l) = 0 \text{ and } I_L(l) = I_0 e^{-\varepsilon_L cl}/2, \quad \text{(for LCPL)} \quad \text{(S21)}$$
$$I_L(l) = 0 \text{ and } I_R(l) = I_0 e^{-\varepsilon_R cl}/2. \quad \text{(for RCPL)}$$

The scattering contributions (S4) from particular volume element *dl* are then

$$dI_R^s = bI_L(l)dl = bI_0 e^{-\varepsilon_L cl}/2dl \text{ and } dI_L^s = aI_L(l)dl = aI_0 e^{-\varepsilon_L cl}/2dl \text{ (for LCPL)} \quad \text{(S22)}$$
$$dI_R^s = aI_R(l)dl = aI_0 e^{-\varepsilon_R cl}/2dl \text{ and } dI_L^s = bI_R(l)dl = bI_0 e^{-\varepsilon_R cl}/2dl \text{ (for RCPL).}$$

Total contributions to the Raman scattering $dI_{Ram}^s(l) = dI_R^s + dI_L^s$ are

$$dI_{Ram}^s(l)_{LCPL} = (b+a)e^{-\varepsilon_L cl} I_0 dl/2 \quad \text{(LCPL)} \quad \text{(S23)}$$
$$dI_{Ram}^s(l)_{RCPL} = (a+b)e^{-\varepsilon_R cl} I_0 dl/2 \quad \text{(RCPL)}$$

and after reaching the point *l* = 0 where the light leaves the sample

$$dI_{Ram}^s(0)_{LCPL} = (be^{-\varepsilon_R' cl} + ae^{-\varepsilon_L' cl})e^{-\varepsilon_L cl} I_0 dl/2 \quad \text{(S24)}$$
$$dI_{Ram}^s(0)_{RCPL} = (ae^{-\varepsilon_R' cl} + be^{-\varepsilon_L' cl})e^{-\varepsilon_R cl} I_0 dl/2.$$

Neglecting the chirality by using $\varepsilon_L' \cong \varepsilon_R' = \varepsilon'$, $\varepsilon_L \cong \varepsilon_R = \varepsilon$,

$$dI_{Ram}^s(0)_{LCPL} = dI_{Ram}^s(0)_{RCPL} = (b+a)e^{-(\varepsilon+\varepsilon')cl} I_0 dl/2 \quad \text{(S25)}$$
$$I_{Ram} = \frac{a+b}{2c} \cdot \frac{1-e^{-(\varepsilon+\varepsilon')cL}}{\varepsilon+\varepsilon'} I_0$$

i.e. it is same for LCPL and RCPL, and analogous to eqs. (S7) and (S8).

However, looking at the difference, we obtain the ICP ROA intensity

$$dI_{ROA}^{ICP}(0) = dI_{Ram}^s(0)_{RCPL} - dI_{Ram}^s(0)_{LCPL} \quad \text{(S26)}$$
$$= (a + be^{-\Delta\varepsilon' cl} - be^{-\Delta\varepsilon cl} - ae^{-(\Delta\varepsilon+\Delta\varepsilon')cl})e^{-(\varepsilon'+\varepsilon)cl} I_0 dl/2$$

and its integral value

$$I_{ROA}^{ICP} = (a\frac{1-e^{-(\varepsilon'+\varepsilon)cL}}{\varepsilon'+\varepsilon} + b\frac{1-e^{-(\varepsilon'+\varepsilon+\Delta\varepsilon')cL}}{\varepsilon'+\varepsilon+\Delta\varepsilon'} - a\frac{1-e^{-(\varepsilon'+\varepsilon+\Delta\varepsilon+\Delta\varepsilon')cL}}{\varepsilon'+\varepsilon+\Delta\varepsilon+\Delta\varepsilon'} - b\frac{1-e^{-(\varepsilon'+\varepsilon+\Delta\varepsilon)cL}}{\varepsilon'+\varepsilon+\Delta\varepsilon})\frac{I_0}{2c}. \quad \text{(S27)}$$

Similarly as before, exploring $\Delta\varepsilon << \varepsilon$, $\Delta\varepsilon' << \varepsilon'$, and $(\Delta\varepsilon' + \Delta\varepsilon)cL << 1$, we get

$$I_{ROA}^{ICP} = [a(\Delta\varepsilon + \Delta\varepsilon') + b(\Delta\varepsilon - \Delta\varepsilon')]\frac{1 - e^{-(\varepsilon'+\varepsilon)cL}[1 + (\varepsilon'+\varepsilon)cL]}{(\varepsilon'+\varepsilon)^2})\frac{I_0}{2c} \quad \text{(S28)}$$

$$CID_{ROA}^{ICP} = [\Delta\varepsilon + DOC\Delta\varepsilon']\frac{1 - e^{-(\varepsilon'+\varepsilon)cL}[1 + (\varepsilon'+\varepsilon)cL]}{(\varepsilon'+\varepsilon)(1 - e^{-(\varepsilon+\varepsilon')cL})})$$

ICP ROA thus has a component (proportional to the difference $\Delta\varepsilon - \Delta\varepsilon'$) that is opposite to SCP ROA.

Detected ROA, DCP. For dual circular polarization schemes, both input and output polarizations are controlled. As for ICP ROA, the sample is irradiated exclusively with L or R CPL, and we can start from, eq. (S21). Using (S4), scattering contributions from particular volume element *dl* are

$$dI_L^s = aI_0 e^{-\varepsilon_L cl} dl/2 \text{ and } dI_R^s = bI_0 e^{-\varepsilon_L cl} dl/2 \text{ (for LCPL)} \quad \text{(S29)}$$
$$dI_R^s = aI_0 e^{-\varepsilon_R cl} dl/2 \text{ and } dI_L^s = bI_0 e^{-\varepsilon_R cl} dl/2 \text{ (for RCPL).}$$

After reaching the point *l* = 0 where the light leaves the sample, they contribute by

$$dI_L^s(0)_{LCPL} = ae^{-(\varepsilon_L' + \varepsilon_L)cl} I_0 dl/2 \text{ and } dI_R^s(0)_{LCPL} = bI_0 e^{-(\varepsilon_L + \varepsilon_R')cl} dl/2 \quad \text{(S30)}$$
$$dI_R^s(0)_{RCPL} = ae^{-(\varepsilon_R' + \varepsilon_R)cl} I_0 dl/2 \text{ and } dI_L^s(0)_{RCPL} = bI_0 e^{-(\varepsilon_R + \varepsilon_L')cl} dl/2.$$

Neglecting the chirality ($\varepsilon_L' \cong \varepsilon_R' = \varepsilon'$, $\varepsilon_L \cong \varepsilon_R = \varepsilon$) gives Raman intensities

(DCPI:)
$$dI_{Ram}^s(0) = dI_{:R}^s(0)_{RCPL} + dI_{:L}^s(0)_{LCPL} = ae^{-(\varepsilon+\varepsilon')cl} I_0 dl \quad \text{(S31)}$$
$$I_{Ram}^s(0) == a\frac{1 - e^{-(\varepsilon+\varepsilon')cL}}{(\varepsilon + \varepsilon')c} I_0$$

(DCPII:)
$$dI_{Ram}^s(0) = dI_{:L}^s(0)_{RCPL} + dI_{:R}^s(0)_{LCPL} = be^{-(\varepsilon+\varepsilon')cl} I_0 dl.$$
$$I_{Ram}^s(0) == b\frac{1 - e^{-(\varepsilon+\varepsilon')cL}}{(\varepsilon + \varepsilon')c} I_0$$

The ROA intensity is

(DCPI:)
$$\begin{aligned} dI_{ROA}^s(0) &= dI_{:R}^s(0)_{RCPL} - dI_{:L}^s(0)_{LCPL} \\ &= a(e^{-(\varepsilon_R' + \varepsilon_R)cl} - e^{-(\varepsilon_L' + \varepsilon_L)cl}) I_0 dl/2 \\ &\cong a(e^{-(\varepsilon'+\varepsilon)cl} - e^{-(\varepsilon'+\varepsilon+\Delta\varepsilon'+\Delta\varepsilon)cl}) I_0 dl/2 \end{aligned} \quad \text{(S32)}$$

(DCPII:)
$$\begin{aligned} dI_{ROA}^s(0) &= dI_{:L}^s(0)_{RCPL} - dI_{:R}^s(0)_{LCPL} \\ &= b(e^{-(\varepsilon_R + \varepsilon_L')cl} - e^{-(\varepsilon_L + \varepsilon_R')cl}) I_0 dl/2 \quad , \\ &\cong b(e^{-(\varepsilon+\varepsilon')cl} - e^{-(\varepsilon+\varepsilon'+\Delta\varepsilon-\Delta\varepsilon')cl}) I_0 dl/2 \end{aligned}$$

and the integral values

$$. I_{ROA}^{DCPI} = (\Delta\varepsilon + \Delta\varepsilon')\frac{1-e^{-(\varepsilon'+\varepsilon)cL}[1+cL(\varepsilon'+\varepsilon)]}{(\varepsilon'+\varepsilon)^2}\frac{aI_0}{2c} \quad \text{(S33)}$$

$$CID_{ROA}^{DCPI} = (\Delta\varepsilon + \Delta\varepsilon')\frac{1-e^{-(\varepsilon'+\varepsilon)cL}[1+cL(\varepsilon'+\varepsilon)]}{2(1-e^{-(\varepsilon+\varepsilon')cL})(\varepsilon'+\varepsilon)}$$

$$I_{ROA}^{DCPII} = (\Delta\varepsilon - \Delta\varepsilon')\frac{1-e^{-(\varepsilon'+\varepsilon)cL}[1+cL(\varepsilon'+\varepsilon)]}{(\varepsilon'+\varepsilon)^2}\frac{bI_0}{2c}.$$

$$CID_{ROA}^{DCPII} = (\Delta\varepsilon - \Delta\varepsilon')\frac{1-e^{-(\varepsilon'+\varepsilon)cL}[1+cL(\varepsilon'+\varepsilon)]}{2(1-e^{-(\varepsilon+\varepsilon')cL})(\varepsilon'+\varepsilon)}$$

Among different ROA modes, we get the relations

$$I_{ROA}^{ICP} = I_{ROA}^{DCPI} + I_{ROA}^{DCPII}, \quad \text{(S34)}$$

$$I_{ROA}^{SCP} = I_{ROA}^{DCPI} - I_{ROA}^{DCPII},$$

$$I_{ROA}^{DCPI} = \frac{1}{2}\left(I_{ROA}^{SCP} + I_{ROA}^{ICP}\right), \text{ etc.}$$

In the present study, only the SCP experiment reported, whereas preliminary results obtained for the other ICP, DCPI and DCPII polarization schemes will be developed in the future.

Experimental Section

The Cu^{II} *R*-3-benzoylcamphorate complex **CuB** was prepared from *R*-camphor following ref. [4]. The *R*- and *S*-bis(3-trifluoroacetyl-camphorate) copper (II) complex, **Cu**, and the bis(pyrrol-2-ylmethyleneamine)-cyclohexane nickel (II), **Ni**, were synthesized and purified based on refs. [5] and [6]. Raman and ROA spectra were measured on a ChiralRaman-2X™ spectrometer of BioTools, operating within 90-2100 cm^{-1}, and on a spectrometer developed at the Palacký University, Olomouc, operating within 45-4000 cm^{-1}, both using 532 nm laser excitation. For ROA measurements with the static magnetic field of 1.5 tesla magnetic cell composed of four neodymium magnets described in ref. [7] was used. The concentration of **CuO** was 2×10^{-4} M in chloroform for the magnetic ROA measurement. In the measurement of natural ROA in the double cell experiments concentrations of *R*-**Cu** and *R*-**CuB** in chloroform were 0.04 M and 0.01 M, respectively. Two identical fused silica cells of 2 mm path length positioned next to each other were used. Concentrations of *R*/*S*-**Cu** and *R*/*S*-**Ni** solutions in ROA measurements were 0.09 M and 0.04 M, respectively.

ECD (*R*-**CuB**) and MCD (**CuO**) spectra were measured on a JASCO J-815 spectrometer; for MCD, a 1.5 tesla permanent magnet was employed. The samples were contained in a rectangular quartz cell of 2 mm optical path length. ECD spectra of **R-CuB** (0.008 M) were recorded within 200-800 nm, MCD of **CuO** (5×10^{-6} M) was measured for both magnet orientations within 300-700 nm. ECD spectra of **Cu** (0.012 M) and **Ni** (0.003 M) were measured using an Olis DSM 17 CD spectrophotometer in the 400-700 nm region. A circular cell of 1 mm path length was used.

On the basis of the ECD spectra, chloroform experimental vibrational frequencies and *DOC* factors (Table S1) induced solvent ROA signal was simulated using our software based on formulas (S13) and (S18). We simulate the difference $I_R - I_L = CID\times(I_R + I_L)$, not the CID itself, as the ratio is ill-defined for regions of zero scattering. Note that absolute ROA/Raman intensities could not be measured; the *y*-scales in the simulation was chosen to fit the experiment.

References

[1] L. D. Barron, *Molecular Light Scattering and Optical Activity*, Cambridge University Press, Cambridge, UK, **2004**.

[2] L. Nafie, *Vibrational optical activity: Principles and applications*, Wiley, Chichester, **2011**.

[3] G. Placzek, in *Handbuch der Radiologie, Vol. 6* (Ed.: E. Marx), Akademische Verlagsgesellschaft, Leipzig, **1934**, p. 205.

[4] T. Wu, X. You, *J. Phys. Chem. A* **2012**, *116*, 8959-8964.

[5] K. M. Nicholas, *Inorg. Chim. Acta* **1976**, *20*, L42.

[6] X. F. Shan, D. H. Wang, C. H. Tung, L. Z. Wu, *Tetrahedron* **2008**, *64*, 5577-5582.

[7] J. Šebestík, J. Kapitán, O. Pačes, P. Bouř, *Angew. Chem. Int. Ed.* **2016**, *55*, 3504-3508.

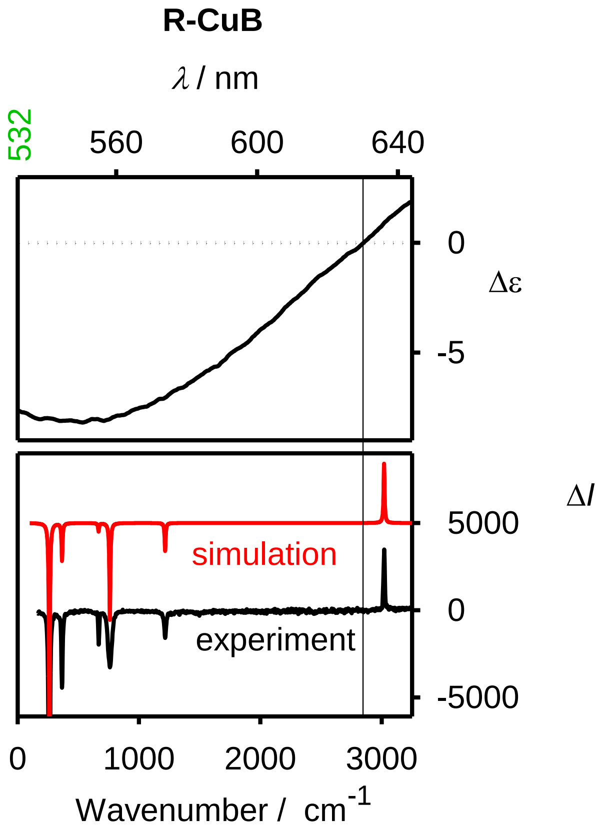

Figure S3. ECD and chloroform-induced ROA spectra of *R*-**CuB** complex. Experimental frequencies and *DOC* factors from **Table S1** were used for the simulation plotted in arbitrary units.

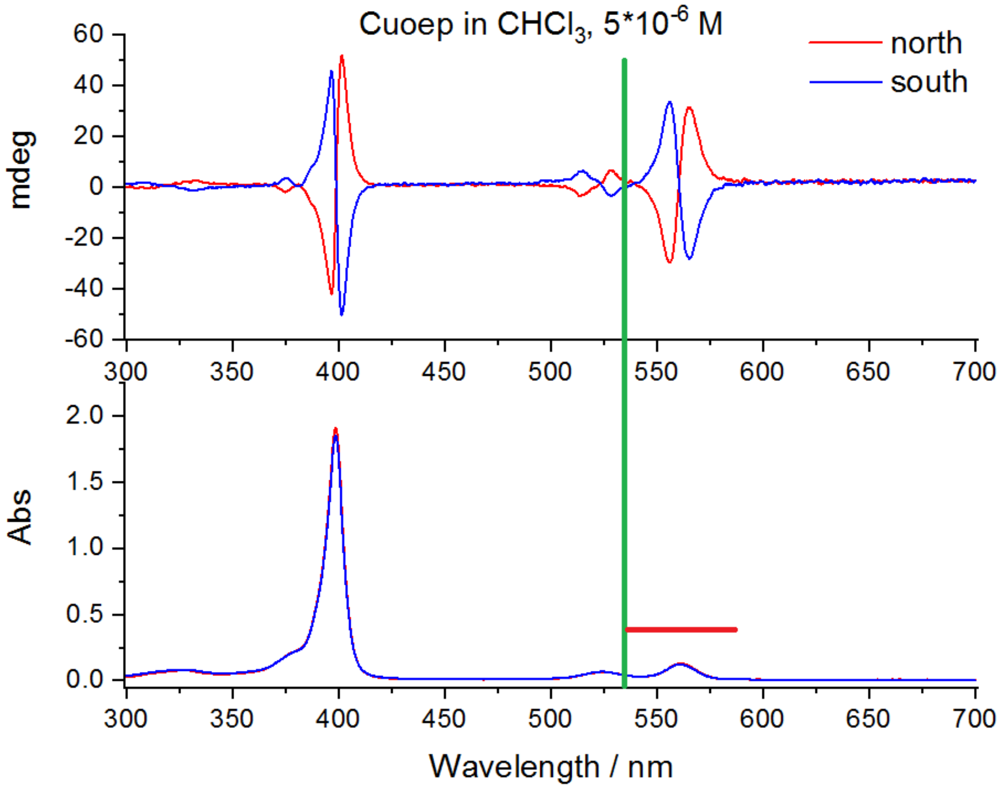

Figure S4. Magnetic CD of the **CuO** complex in chloroform, the red bar indicates approximate extent of the Raman spectrum (100-2100 cm^{-1}).

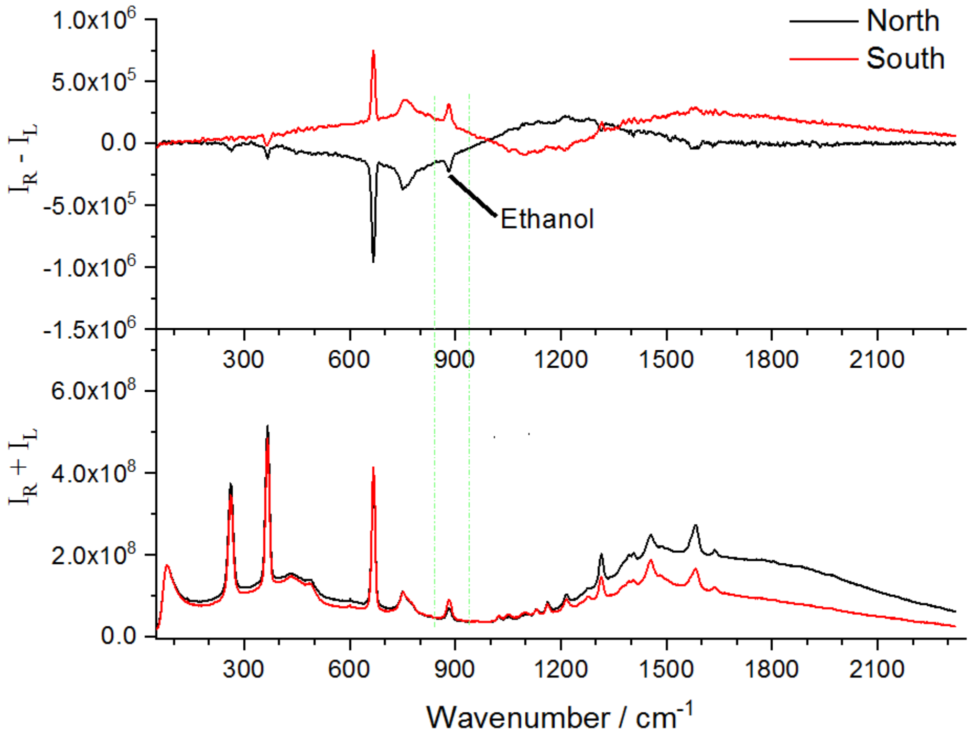

Figure S5. Magnetic ROA (top) and Raman (bottom) spectra obtained with ethanol (solvent I) and **CuO** in $CHCl_3$ (solvent II) in the double-cell experiment.

Table S1. Selected Spectral Parameters of chloroform vibrational transitions: frequency (ω/cm^{-1}), Raman intensity (I_0/a.u.), depolarization ratio (p), and Degree of Circularity (*DOC*). The calculated results were obtained at the B3LYP/aug-cc-pVTZ/CPCM level.

Degeneracy	$\omega_{Cal.}$	$\omega_{Exp.}$	$I_{0, Cal.}$	$p_{, Cal.}$	$DOC_{Cal.}$	$DOC_{Exp.}$
2x	256	261	17	0.75	0.71	0.69
1x	367	365	57	0.11	-0.60	-0.75
1x	659	667	111	0.03	-0.90	-0.99
2x	710	760	34	0.75	0.71	0.54
2x	1233	1215	16	0.75	0.71	0.66
1x	3183	3019	415	0.18	-0.40	-0.59

Comment 1.

From (S3) we want to find extreme of the function $\Delta I(l)=I_R-I_L=\frac{I_0}{2}(e^{-\varepsilon_R cl}-e^{-\varepsilon_L cl})$. Therefore, we set its first derivative to zero

$$\frac{\partial \Delta I}{\partial l}=\frac{I_0 c}{2}(-\varepsilon_R e^{-\varepsilon_R cl}+\varepsilon_L e^{-\varepsilon_L cl})=0$$

which gives $\varepsilon_R/\varepsilon_L=e^{-(\varepsilon_L-\varepsilon_R)cl}$. Using $\Delta\varepsilon=\varepsilon_L-\varepsilon_R$, we get $\varepsilon_R/\varepsilon_L=e^{-\Delta\varepsilon cl}$, $\ln(\varepsilon_R/\varepsilon_L)=-\Delta\varepsilon cl$, and finally $l_{\max}=\frac{\ln(\varepsilon_L/\varepsilon_R)}{\Delta\varepsilon c}$. In the limit of weak dichroism, $|\Delta\varepsilon| << |\varepsilon_R|$, we get an approximate value $l_{\max}=\frac{\ln[(\varepsilon_R+\Delta\varepsilon)/\varepsilon_R]}{\Delta\varepsilon c}=\frac{\ln(1+\Delta\varepsilon/\varepsilon_R)}{\Delta\varepsilon c}\cong\frac{1}{c\varepsilon}$.

Using $l_{\max}=\frac{\ln(\varepsilon_L/\varepsilon_R)}{\Delta\varepsilon c}$, we arrive at the maximal intensity difference

$$\Delta I(l_{\max})=\frac{I_0}{2}(e^{-c\varepsilon_R\frac{\ln(\frac{\varepsilon_L}{\varepsilon_R})}{\Delta\varepsilon c}}-e^{-c\varepsilon_L\frac{\ln(\frac{\varepsilon_L}{\varepsilon_R})}{\Delta\varepsilon c}})=\frac{I_0}{2}(e^{-\frac{\varepsilon_R}{\Delta\varepsilon}\ln(\frac{\varepsilon_L}{\varepsilon_R})}-e^{-\frac{\varepsilon_L}{\Delta\varepsilon}\ln(\frac{\varepsilon_L}{\varepsilon_R})})=\frac{I_0}{2}(e^{-\frac{\varepsilon_L-\Delta\varepsilon}{\Delta\varepsilon}\ln(1+\frac{\Delta\varepsilon}{\varepsilon_R})}-e^{-\frac{\varepsilon_L}{\Delta\varepsilon}\ln(1+\frac{\Delta\varepsilon}{\varepsilon_R})})$$

$=\frac{I_0}{2}(e^{\ln(1+\frac{\Delta\varepsilon}{\varepsilon_R})}-1)e^{-\frac{\varepsilon_L}{\Delta\varepsilon}\ln(1+\frac{\Delta\varepsilon}{\varepsilon_R})}\cong\frac{I_0}{2}\frac{\Delta\varepsilon}{e\varepsilon_R}$. Using $l_{\max}=\frac{1}{c\varepsilon}$ provides the same result faster,

$\Delta I(l_{\max})=\frac{I_0}{2}(e^{-\frac{\varepsilon_R}{\varepsilon}}-e^{-\frac{\Delta\varepsilon+\varepsilon_R}{\varepsilon}})\cong\frac{I_0}{2}\frac{\Delta\varepsilon}{e\varepsilon}$. (A common error during this derivation appears to be interchanging the two limits, $e^{-\frac{\varepsilon_R}{\varepsilon}}\to\frac{1}{e}$ and $e^{-\frac{\Delta\varepsilon}{\varepsilon}}\to 1-\frac{\Delta\varepsilon}{\varepsilon}\sim 1$.)

Comment 2.

One has to be also careful when evaluating the weak absorption limit ($cL(\varepsilon'+\varepsilon)<<1$) of (S13). Using directly $[cL(\varepsilon'+\varepsilon)+1]\sim 1$ in $CID=\frac{a(\Delta\varepsilon+\Delta\varepsilon')+b(\Delta\varepsilon'-\Delta\varepsilon)}{a+b}\frac{1-e^{-(\varepsilon'+\varepsilon)cL}[cL(\varepsilon'+\varepsilon)+1]}{2(\varepsilon'+\varepsilon)(1-e^{-(\varepsilon'+\varepsilon)cL})}$ gives

$CID=\frac{a(\Delta\varepsilon+\Delta\varepsilon')+b(\Delta\varepsilon'-\Delta\varepsilon)}{a+b}\frac{1}{2(\varepsilon'+\varepsilon)}$, which is a wrong result. Expanding the exponential factor up to the second order $e^{-(\varepsilon'+\varepsilon)cL}\cong 1-(\varepsilon'+\varepsilon)cL+(\varepsilon'+\varepsilon)^2c^2L^2/2$ allows us to arrive to the true limit:

$$CID\cong\frac{a(\Delta\varepsilon+\Delta\varepsilon')+b(\Delta\varepsilon'-\Delta\varepsilon)}{a+b}\frac{1-[1-(\varepsilon'+\varepsilon)cL+(\varepsilon'+\varepsilon)^2c^2L^2/2][cL(\varepsilon'+\varepsilon)+1]}{2(\varepsilon'+\varepsilon)(\varepsilon'+\varepsilon)cL}$$

$$\cong\frac{a(\Delta\varepsilon+\Delta\varepsilon')+b(\Delta\varepsilon'-\Delta\varepsilon)}{a+b}\frac{1-1+(\varepsilon'+\varepsilon)cL-(\varepsilon'+\varepsilon)^2c^2L^2/2-cL(\varepsilon'+\varepsilon)+(\varepsilon'+\varepsilon)cLcL(\varepsilon'+\varepsilon)}{2(\varepsilon'+\varepsilon)(\varepsilon'+\varepsilon)cL}$$

$$\cong\frac{a(\Delta\varepsilon+\Delta\varepsilon')+b(\Delta\varepsilon'-\Delta\varepsilon)}{a+b}\frac{cL}{4}.$$
